# Conservatism predicts aversion to consequential Artificial Intelligence

**DOI:** 10.1371/journal.pone.0261467

**Published:** 2021-12-20

**Authors:** Noah Castelo, Adrian F. Ward

**Affiliations:** 1 Department of Marketing, Business Economics, and Law, University of Alberta, Edmonton, Alberta, Canada; 2 Department of Marketing, University of Texas at Austin, Austin, Texas, United States of America; St John’s University, UNITED KINGDOM

## Abstract

Artificial intelligence (AI) has the potential to revolutionize society by automating tasks as diverse as driving cars, diagnosing diseases, and providing legal advice. The degree to which AI can improve outcomes in these and other domains depends on how comfortable people are trusting AI for these tasks, which in turn depends on lay perceptions of AI. The present research examines how these critical lay perceptions may vary as a function of conservatism. Using five survey experiments, we find that political conservatism is associated with low comfort with and trust in AI—i.e., with AI aversion. This relationship between conservatism and AI aversion is explained by the link between conservatism and risk perception; more conservative individuals perceive AI as being riskier and are therefore more averse to its adoption. Finally, we test whether a moral reframing intervention can reduce AI aversion among conservatives.

## Introduction

Artificial intelligence (AI) has the potential to revolutionize society, with impact ranging from the broadest reaches of industry and policy to the minutiae of daily life. Recent progress in AI research has enabled computers to perform tasks traditionally thought of as requiring human intelligence. Today, AI can drive cars by accurately tracking the environment and making decisions in real time; it can diagnose diseases and make treatment recommendations based on a comprehensive understanding of all published medical research; it can even teach itself how to play complex games that require strategic planning. In all of these cases and many more it can match or outperform even the most expert humans [1–4]. AI has the potential to improve outcomes for many people, in many domains, and indeed is already doing so. However, the benefits promised by AI are contingent on people accepting and using this technology—and acceptance is far from guaranteed.

If people are averse to relying on AI, the ability of this technology to positively impact society as a whole could be hindered. For example, if AI can outperform humans at safely driving cars but people are averse to relying on AI for this task, traffic deaths might remain higher than they would be if driverless cars were more readily embraced. More generally, active distrust or aversion towards AI could impact the continued technical development and funding of AI research, as well as the public policies that are created to regulate it, which could further curtail the technology’s ability to positively impact society.

In this research, we first show that political conservatism is a strong predictor of AI aversion and then test interventions that can reduce this aversion. As an individual difference factor, conservatism is both psychologically meaningful and practically relevant. At a psychological level, conservatism is not just about how one votes; it is about how one perceives the world. Conservatives possess a heightened sensitivity to threat and uncertainty [5,6]; we find that this sensitivity (operationalized in terms of perceived risk) is responsible for the link between conservatism and AI aversion. The potential consequences of this association are far-reaching. For example, more positive perceptions of AI among liberals could lead liberal states and citizens to adopt AI-based technologies more quickly than their conservative counterparts, and therefore to reap disproportionate benefits from those technologies. In addition to these potentially inequality-inducing dynamics, ideological or partisan gridlock on the issue of regulating AI could hamper effective and much-needed national legislation on issues such as autonomous vehicles and the use algorithms in hiring and sentencing decisions. Understanding ideological sources of aversion to AI is therefore an important task for psychologists.

### Aversion to algorithms and AI

An artificially intelligent agent is something that receives information from the environment and uses that information to perform actions [7]. How do people perceive these technologies, and how do those perceptions influence the degree to which people are willing to use AI? The answers to these questions are important because AI’s ability to positively impact society is partly a function of people’s adoption and use. Note that our research focuses on specific applications of AI such as driverless cars and automated medical diagnoses—often referred to as “artificial narrow intelligence,” as opposed to a hypothetical AI that has human-like abilities in all areas, often referred to as “artificial general intelligence.”

The literature on individual’s perceptions and adoption of AI (and of algorithms more generally) generally shows that humans prefer to rely on other humans rather than on algorithms to make decisions [8,9]. This tendency has been documented in many domains, such as admitting MBA students [10], hiring employees [11], receiving medical treatment [12], and forecasting stock prices [13]—despite the fact that algorithms outperform humans in these and many other domains [14]. Furthermore, this tendency exists when people actively decide whether to rely on algorithms after seeing them perform [15] as well as when people consider algorithmic decision-making in prospect (i.e., in hypothetical scenarios) [16]. This literature suggests that people may be unlikely to rely on and trust AI as much as they would a human in many domains. Although some research has identified situations in which people actually prefer to rely on algorithms more than on humans [17], this remains an exception rather than the rule in the literature.

More recent research has begun to explore potential moderators of this aversion to relying on automated forms of decision making (including AI). Research has shown that trust in algorithms can be increased by giving people a small degree of control over the algorithm’s output [15] or by explaining how the algorithm works [18]. Furthermore, algorithm aversion is lower for tasks that seem highly objective (vs. subjective) in nature [8]. Our research contributes to this stream of work, specifically focusing on how individual differences—in this case, ideological conservatism—impact perceptions of a technology with the proven potential to transform people’s lives.

### Conservatism and risk

Conservatism has been defined as “the tendency to prefer safe, traditional and conventional forms of institutions and behavior” [19]. In the most basic sense, conservatism is associated with a preference for tradition and stability, whereas liberalism is associated with a preference for innovation and reform [20]. Supporting these classical conceptions of conservatism, a more recent meta-analysis proposed the uncertainty-threat model of political conservatism, which states that conservatism consists of resistance to change and opposition to equality, both of which serve a fundamental need to reduce threat and uncertainty [5,6].

This conceptualization is supported by research indicating that conservatism is fundamentally associated with a greater focus on negativity, fear, and risk. A wealth of research has demonstrated that conservatives react more strongly than liberals to environmental stimuli that are uncertain, ambiguous, or unexpected in nature, across both psychological and physiological measures of reactivity [21]. This tendency is especially pronounced for stimuli that are negative or potentially threatening [22]. For example, compared to liberals, conservatives have stronger physiological reactions (measured by electrical skin conductance) to negatively valenced stimuli [23], are more likely to perceive emotionally ambiguous faces as threatening [24], and are more likely to believe warnings about potential hazards [25]. This research suggests that conservatism reflects a fundamental mindset that structures how people perceive and interact with the world around them, and that a heightened sensitivity to risk lies at the heart of this mindset.

Note that conservatism can be broken down into social and fiscal conservatism or considered as a more general conservatism—we focus primarily on social conservatism in our studies as this aspect has received the most attention in psychological research, where conservatism is explicitly conceptualized as a form of *social* cognition [6].

### Sources of perceived risk

Several prominent conceptions of risk perception share the central idea that perceived risk is jointly determined by two factors: the uncertainty of an outcome, and the importance of that outcome’s consequences. The classic consequentialist perspective holds that something’s perceived risk is a function of the *importance of its potential consequences*, multiplied by the *likelihood of those consequences occurring* [26,27]. For example, having a complex surgery has very important potential consequences (i.e., a surgical error leading to disability or death), which makes the surgery potentially risky; however, the overall perceived risk of that surgery will also depend on how likely a surgical error is perceived to be (i.e., on the skill and track record of the surgeon). More recent approaches have found that perceived risk varies along the similar dimensions of *dread*, which is affective and concerns perceived “catastrophic potential,” and *risk of the unknown*, which is a more cognitive assessment of probabilities [28,29]. For example, nuclear war is highly dreaded, but the perceived probability that it will occur is relatively low [29].

This research examines how the dread component of risk, which parallels the *importance of potential consequences* component, may amplify political differences in AI aversion for particularly consequential tasks. Our definition of consequentialness parallels the classic definition of this concept in the risk perception literature: a task is more consequential if failing the task has serious or significant consequences, which can include financial, physical, social, or psychological consequences. For example, using AI to control driverless cars clearly has more important potential consequences than using AI to recommend a movie on Netflix; the risk of death is more consequential than the risk of watching a boring movie. In this research, we use between-task variations in consequentialness to examine how consequentialness may amplify political differences in perceived risk—and, consequently, in willingness to rely on AI.

### The present research

**Fig 1:**
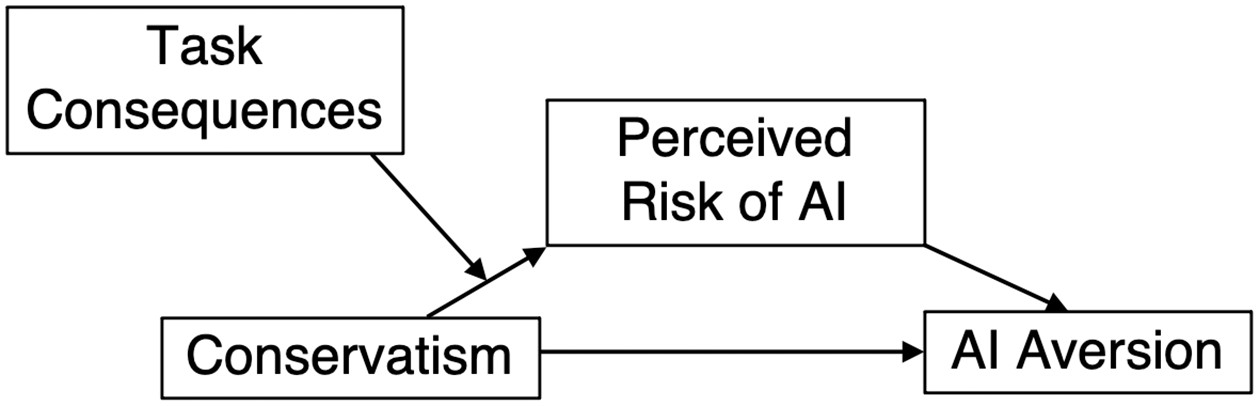
A conceptual model of Conservatism’s effects on AI aversion.

The preceding discussions of AI, conservatism, and risk suggest a conceptual model of how conservatism might affect aversion to AI, which we depict below (see Fig 1). Because conservatism is associated with greater propensity to perceive risk, we expect that more conservative people will be more likely to see AI as risky. Because one of the major components of perceived risk is the consequentialness of potential outcomes, we expect that the consequentialness of the task that AI is being used for will moderate the effect of conservatism on perceived risk, such that the effect will be eliminated for tasks that are relatively inconsequential. Finally, we expect that the perceived riskiness of AI will mediate the effect of conservatism on individuals’ aversion to AI.

This model guides our research into how a common psychological variable affects people’s attitudes towards a technology with perhaps unparalleled potential to improve outcomes across countless domains. Our focus on individual differences contrasts with previous approaches to understanding reliance on algorithms, allowing us to explain some of the heterogeneity in responses to emerging technologies and suggesting potential interventions for changing those attitudes.

We test this model in 3 studies. The primary relationship that we focus on in the paper is the relationship between conservatism and AI aversion. We operationalize aversion as comfort relying on AI in our first study (such that lower comfort reflects greater AI aversion), and as trust in AI in subsequent studies (such that lower trust reflects greater AI aversion). Study 1 provides initial evidence for this relationship and shows that perceived risk plays a mediating role. Study 2 shows that this pattern holds for consequential tasks, but not for inconsequential tasks. Study 3 tests the full predicted pattern of moderated mediation: conservatism predicts trust in AI because it predicts risk; because risk is less relevant for inconsequential tasks, the divide between liberals and conservatives only exists for consequential tasks. Finally, Studies 4 and 5 test whether a moral reframing intervention [30,31] can reduce AI aversion among conservatives without explicitly attempting to reduce perceived risk. In all studies, we report all measures, manipulations and exclusions. Sample sizes for each study were determined prior to any data analysis.

## Study 1

In our first study, we examine how conservatism affects AI aversion. We operationalize AI aversion by asking participants how comfortable they would feel relying on AI for two tasks. We focus our examination on two particularly promising (and consequential) applications of AI: for self-driving cars, and for diagnosing diseases. These applications were validated to be high in perceived consequentialness in the pretest to Study 2 (see Table 1). We test the effect of conservatism on trust in AI both in isolation and alongside other potentially important demographic factors (e.g., income, education level). We then test for mediation via our proposed mechanism: perceived risk.

**Table 1:** Direct and indirect effects of social conservatism on perceived risk of and comfort with AI (standardized *β* coefficients).

Effect	Risk	Total	Comfort	
			Direct	Indirect
Path	*a*	*c*	*c*’	*b*
Medical Diagnosis	.13**	-.12*	-.04	-.67**
Driverless Cars	.21**	-.20**	0.03	-.76**
In General	.06	-.14**	-.09*	-.58**

Note: Age, gender, education, and income are included as covariates in the mediation models reported above.

^†^ = *p* < .10

* = *p* < .05

** = *p* < .01.

### Method

400 MTurk users (44% female, mean age = 35.3) read the following introduction to AI (see Appendix for complete stimuli):

“*Every year, Artificial Intelligence (AI) becomes capable of performing new tasks that only humans could do before, from beating chess grandmasters and the best human players on the game show Jeopardy!, to driving cars and diagnosing diseases*.

*Ongoing developments in Artificial Intelligence include the creation of systems intended to handle increasingly complex tasks—for example, reading medical journals, diagnosing diseases, delivering treatment recommendations, and even controlling automated driverless cars*.”

In this first study, we also wanted to ensure that participants were sufficiently engaged with the material and were thoughtfully considering the technology and its implications. In addition to the basic introduction to AI, which describes some of the technology’s benefits, we therefore also described some of the potential risks associated with AI. Note that future studies replicate the results of this study without explicitly describing these risks. Participants in this study therefore also read the following:

“*Many people are worried that developing AI is risky for several reasons. For example, AI can already do many jobs better than humans can, and the number of such jobs is growing all the time—including jobs like truck drivers and fast food workers and even some kinds of lawyers and doctors. One risk is therefore that developing AI will put many humans out of a job*.

*Another risk that people worry about is that AI could potentially become even more intelligent than humans and start to develop its own goals. This could mean that AI decides humans are no longer helpful for its development and could start to manipulate or hurt humans*.”

Participants then reported their perceptions of how risky it is to rely on AI for “medical tasks such as diagnosing diseases and providing treatment recommendations” and for “controlling driverless cars” and how comfortable they would be relying on AI for these tasks. We also asked participants how risky it is to rely on AI in general and how comfortable they are with the idea of relying on AI. Finally, participants reported their social conservatism, fiscal conservatism, and conservatism in general, and reported age, gender, income, and education, which are demographic variables that are sometimes associated with conservatism and may also be associated with responses to new technologies. All measures (except demographics) used 0–100 scales. This research was approved by the IRB at Columbia University. Participants in all studies provided informed consent to participate in the surveys by clicking a button indicating their consent. All data and R code for this article are available at https://osf.io/5wehr/?view_only=7f2ef4c8fcea4c0ea05a706987df9e96.

### Results

We first report results for how social conservatism affects comfort relying on AI for specific tasks, since these main effects are our primary focus. We then report how conservatism affects perceived risks of AI, which is our proposed mechanism of the main effects. We then report mediation analyses testing whether perceived risks mediate the effects of conservatism on comfort relying on AI. For each analysis, we report how conservatism affects the dependent variable while controlling for a range of other potentially relevant demographic variables. Tables 2 and 3 display the effects of each of those variables, along with conservatism, on each of our dependent variables. The Web Appendix reports results using general and fiscal conservatism instead of social conservatism.

**Table 2:** Conservatism-Only and Conservatism + Demographics Models of Comfort with AI, Study 1.

Model	Conservatism Only			Conservatism + Demographics		
Domain	Medical Diagnoses	Self -Driving Cars	General	Medical Diagnoses	Self-Driving Cars	General
Conservatism	-.12 (.05)*	-.20 (.05)**	-.14 (.04)**	-.13 (.05)*	-.19 (.05)**	-.15 (.05)**
Education:						
High school				17.13 (17.10)	28.66 (17.49)	34.76 (15.21)*
Some college				11.82 (18.82)	30.53 (17.19)†	31.24 (14.95)*
2-year college				17.96 (17.05)	36.05 (17.43)*	38.89 (15.16)*
4-year college				15.52 (16.75)	32.47 (17.13)†	36.45 (14.89)*
Graduate degree				18.07 (17.27)	33.10 (17.66)†	37.54 (15.35)*
Income				-.01 (.91)	.69 (.93)	-0.53 (.81)
Age				.08 (.14)	-.23 (.14)†	0.04 (.12)
Female				-9.83 (2.97)**	-10.68 (3.03)**	-10.03 (2.63)**
Intercept	40.43 (3.42)*	34.51 (3.56)**	44.47 (3.09)**	36.24 (18.16)*	24.15 (18.62)	24.38 (16.17)
*R* ^*2*^	.01	.04	.02	.05	.09	.07

Gender (“Female”) is dummy coded with “Male” as the reference group.

Education is a dummy coded variable with “less than high school” as the reference group.

^†^ = *p* < .10

* = *p* < .05

** = *p* < .01.

**Table 3:** Conservatism-Only and Conservatism + Demographics Models of Perceived Risk of AI, Study 1.

Model	Conservatism Only			Conservatism + Demographics		
Domain	Medical Diagnoses	Self -Driving Cars	General	Medical Diagnoses	Self-Driving Cars	General
Social Conservatism	.13 (.04)**	.23 (.05)**	.05 (.05)	.14 (.05)**	.22 (.05)**	.08 (.05)
Education:						
High school				-12.47 (15.52)	-21.22 (15.91)	-25.17 (14.98)†
Some college				-17.13 (15.26)	-30.32 (15.64)†	-28.96 (14.72)*
2-year college				-16.77 (15.48)	-33.80 (15.86)*	-31.65 (14.93)*
4-year college				-16.27 (15.20)	-28.72 (15.59)†	-27.25 (14.67)†
Graduate degree				-16.47 (15.67)	-32.51 (16.06)*	-25.97 (15.12)†
Income				-.01 (.91)	-0.46 (.85)	-0.81 (.80)
Age				-0.10 (.12)	0.12 (.13)	-0.19 (.12)
Female				10.97 (2.69)**	10.00 (2.75)**	7.24 (2.59)**
Intercept	67.28 (3.11)**	72.79 (3.22)**	62.12 (2.99)**	72.30 (16.48)**	82.25 (16.89)**	91.01 (15.90)**
*R* ^*2*^	.02	.05	.001	.07	.11	.04

Gender (“Female”) is dummy coded with “Male” as the reference group.

Education variables are dummy coded with “less than high school” as the reference group.

^†^ = *p* < .10

* = *p* < .05

** = *p* < .01.

### Comfort

We first performed a series of OLS regressions assessing the relationship between conservatism and comfort relying on AI (i) for medical diagnoses, (ii) for self-driving cars, and (iii) in general. For each application of AI, we assessed the effect of conservatism both in isolation and alongside other demographic variables that might reasonably predict comfort relying on AI: age, gender, income, and education level. Controlling for these other factors, increasing levels of conservatism predict decreasing comfort with AI for medical applications (β = -.13, *p* = .014), driverless cars (β = -.19, *p* < .001), and in general (β = -.15, *p* = .004). Conservatism and comfort (as well as risk in the next section) were standardized so that beta coefficients represent effect sizes. Power analysis revealed that this sample size had 99% power to detect these effect sizes, and that a sample size of 62 would be required to detect these effect sizes with 80% power. The only other variable to consistently affect comfort was gender: for both uses of AI and for comfort with AI in general, men felt significantly more comfortable than women (see Table 2 for the effects of all demographic variables, noting that the demographic variables are not standardized).

### Risk

Next, we performed a series of OLS regressions assessing the relationship between conservatism and perceived risk associated with AI, controlling for the demographic covariates. We found that increases in conservatism predicted increases in perceived risk of using AI for both specific applications included in this study: medical diagnoses (β = .14, *p* = .005) and driverless cars (β = .22, *p* < .001). The effect of conservatism on perceived risk of AI in general was directionally consistent with the results found for specific applications, but not statistically significant (β = .08, *p* = .112). These regression coefficients are standardized. Again, the only other variable to affect trust was gender: for both uses of AI and for AI in general, men perceived significantly less risk than women. Full results of these analyses are shown in Table 3. Risk, comfort, and conservatism are standardized. As with our analysis of trust in AI, parallel analyses replacing social conservatism with overall conservatism and fiscal conservatism replicated these results (see web appendix for full results).

### Mediation

We assessed the relationship between conservatism, perceived riskiness of AI, and trust in AI in a series of mediation models. We included age, gender, income, and education as covariates in each mediation analysis; parallel analyses excluding these covariates yield nearly identical results (please see web appendix for these analyses). First, we tested whether the perceived risk of using AI for medical applications mediated the total effect of social conservatism on comfort using AI for medical applications (β = -.12, *t*(399) = 2.45, *p* = .016). Using Hayes (2013) PROCESS Model 4, we found evidence of mediation with a significant indirect effect of conservatism on comfort through risk (B = -.08, *SE* = .03, 95% CI = -.15 to -.02) and a non-significant direct effect after controlling for perceived risk (β = -.04, *t*(399) = .97, *p* = .332).

Second, we tested whether the perceived risk of using AI for driverless cars mediated the total effect of social conservatism on comfort using AI for driverless cars (β = -.20, *t*(399) = 4.08, *p* < .001). We once again found evidence of mediation with a significant indirect effect of conservatism on comfort through risk (β = -.16, *SE* = .04, 95% CI = -.23 to -.08) and a non-significant direct effect after controlling for perceived risk (β = -.04, *t*(399) = .95, *p* = .344).

**Fig 2:**
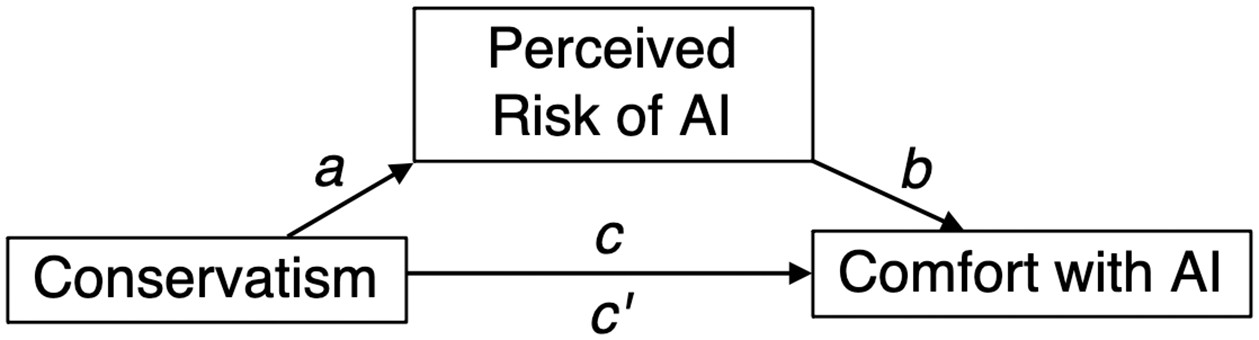
Mediation model and standardized coefficients, Study 1.

Since conservatism did not affect perceived risk of using AI in general, risk did not mediate comfort with AI in general (B = -.03, *SE* = .03, 95% CI = -.09 to .02). Please see Fig 2 for full results. Note that we cannot conclude that conservatism causes risk perception from these data, since we did not manipulate conservatism. Thus, as in most research studying the psychology of conservatism, this relationship is measured in terms of correlation rather than causation. Similarly, the relationship between perceived risk and comfort with AI is also correlational. This is also true for the mediation analysis reported in Study 3. These mediation analyses are therefore compatible with only one of several possible models. Finally, note that while our *R*^*2*^ values are low, suggesting that the independent variables we measure explain only a small portion of the variance in our dependent variables, they are nevertheless above zero and in the normal range for social psychology research [32], therefore suggesting that conservatism in particular is indeed a significant factor in explaining attitudes towards AI.

### Discussion

Study 1 provides evidence that conservatism is associated with aversion to AI. For two potentially transformational applications of AI—medical diagnoses and self-driving cars—increasing conservatism predicts decreasing trust in AI. This relationship cannot be explained by other potentially relevant demographic variables that may be associated with conservatism and/or perceptions of AI, such as age, income, or education level; the conservatism-trust relationship is robust across models that include and exclude these covariates. Rather, results from our mediation analyses suggest that the relationship between conservatism and trust in AI may be attributable to differences in perceived risk. We further explore the potential causal mechanism in Study 2.

Interestingly, we found that the links between conservatism, risk, and trust in AI were stronger when participants considered specific applications of AI than when they thought about AI in general. We suspect that this discrepancy may reflect the fact that many of the tasks for which AI can be used are relatively inconsequential—and therefore possess negligibly low levels of risk. If general considerations of AI bring to mind a mix of applications—including not just advanced medical procedures and self-driving cars, but also music recommendations and smart thermostats—this may undermine the relationship between conservatism and perceived risk, thus weakening the relationship between conservatism and trust. This would be consistent with our theoretically derived prediction that task consequentialness should moderate the relationship between conservatism and perceived risk of AI. We test this hypothesis in Studies 2 and 3.

## Study 2

AI can be used for a wide variety of tasks, and these tasks vary in terms of the importance of the consequences of success and failure. For example, AI can be used to recommend movies and music, or to diagnose diseases and drive cars. The latter tasks are clearly much more consequential than the former. Since perceived risk is partly a function of the importance of consequences [26,29], tasks such as recommending movies and music should seem relatively low risk. Conservatives should therefore be less likely to perceive the use of AI as risky for such tasks, and more likely to feel comfortable relying on AI. Study 2 tests whether the consequentialness of a task does indeed moderate the effect of conservatism on trust in AI. Recall that our proposed mechanism for the relationship between conservatism and trust is perceived risk. Since perceived risk is partially determined by consequentialness, this study therefore tests our theorized process using moderation.

### Method

In Study 2, we ask people to evaluate AI in the context of 27 tasks that AI can already accomplish, with varying levels of consequentialness; these tasks, which range from evaluating jokes to diagnosing diseases, are listed in Table 4 below. We first assessed the perceived consequentialness of these tasks by asking 221 MTurk users to rate each task according to how “consequential” each of the tasks seems, using the following wording: "Please use the sliders to indicate how much each of the tasks below seems to be consequential vs. inconsequential. Consequential means the task is important and doing well has serious consequences. Inconsequential means the task is not very important and doing well doesn’t have serious consequences.” This definition closely parallels the classic conception of the “dread” and the “importance of outcomes” components of perceived risk [26,29]. We then divided these tasks into two groups: those that were rated (1) above and (2) below the scale midpoint (the scale endpoints were labeled “very inconsequential” and “very consequential”). Each task was therefore classified as consequential or inconsequential depending on whether it was rated as above or below the scale midpoint, which allowed us to create a binary measure of consequentialness.

**Table 4:** Tasks used in Study 2.

Diagnose Disease (39)	Rec. Disease Treatment (38)	Fly Plane (35)
Hire & Fire Employees (28)	Drive Car (26)	Drive Subway (25)
Drive Truck (24)	Predict Recidivism (23)	Buy Stocks (22)
Analyze Data (21)	Predict Stock Market (20)	Rec. Marketing Strategy (18)
Pred. Employee Success (17)	Give Directions (15)	Rec. Romantic Partners (14)
Predict Weather (9)	Write News Story (8)	Schedule Events (7)
Predict Elections (6)	Predict Student Success (6)	Rec Gift (2)
Write Song (-13)	Recommend Restaurant (-14)	Recommend Movies (-17)
Recommend Music (-18)	Play Piano (-18)	Evaluate Jokes (-24)

Note: Numbers in parentheses are the tasks’ consequentialness ratings.

Rec. = Recommend; Pred. = Predict.

We then asked 417 MTurk users (44% female, average age = 36.0) to consider using AI to perform each of these 27 tasks; tasks were presented in random order, and participants were not provided with any information about the rated consequentialness of each task. AI was described as follows, without any mention of potential risks:

“*Artificial intelligence (AI) refers to a set of computer programs that can be used to accomplish a task. Thanks to rapid progress in computer science, AI can now be used to accomplish a wide range of tasks that humans would generally do, without being explicitly instructed how to do so by humans*.”

For each task, participants were asked to indicate how much they would trust AI to perform the task (“Please indicate how much you would trust AI to perform each of the tasks below”). As in Study 1, participants also reported their degree of social and fiscal conservatism, plus age and gender (all measures except age and gender used 0–100 scales). Due to constraints on study length, we did not measure perceived risk of using AI for each task in Study 2.

### Results

Because each participant reported trust in AI for each of the 27 tasks, we first converted the data from wide to long format in order to create a variable for “task” and a variable for “trust in AI.” There were therefore 27 rows for each participant (one per task), plus a “participant” variable. Each row was assigned a 1 if it represented a consequential task, and a 0 if it represented an inconsequential task. This binary measure of consequentialism used the scale midpoint as the dividing point. See web appendix for alternative analyses using different dividing points (e.g., +10 and -10 instead of above and below 0), which replicate the results reported here.

We then ran an OLS regression using the binary measure of task consequentialness (coded as 0 for inconsequential and 1 for consequential), social conservatism (standardized), and their interaction to predict trust in AI (standardized), including age, gender, and a participant fixed effect as covariates. This revealed effects for gender, β = .11, *SE* = .02, *p* < .001, such that females trusted AI less than males, age, β = -.02, *SE* = .009 *p* = .011, such that older participants trusted AI less than younger participants, but no effect of participant, β < .001, *SE* < .001, *p* = .972. More pertinent to our theorizing, we observed no main effect of consequentialness, β = .03, *SE* = .02, *p* = .127, no main effect of conservatism, β = .02, *p* = .339; and a significant interaction between conservatism and task consequentialness, β = .06, *SE* = .02, *p* = .003.

**Fig 3:**
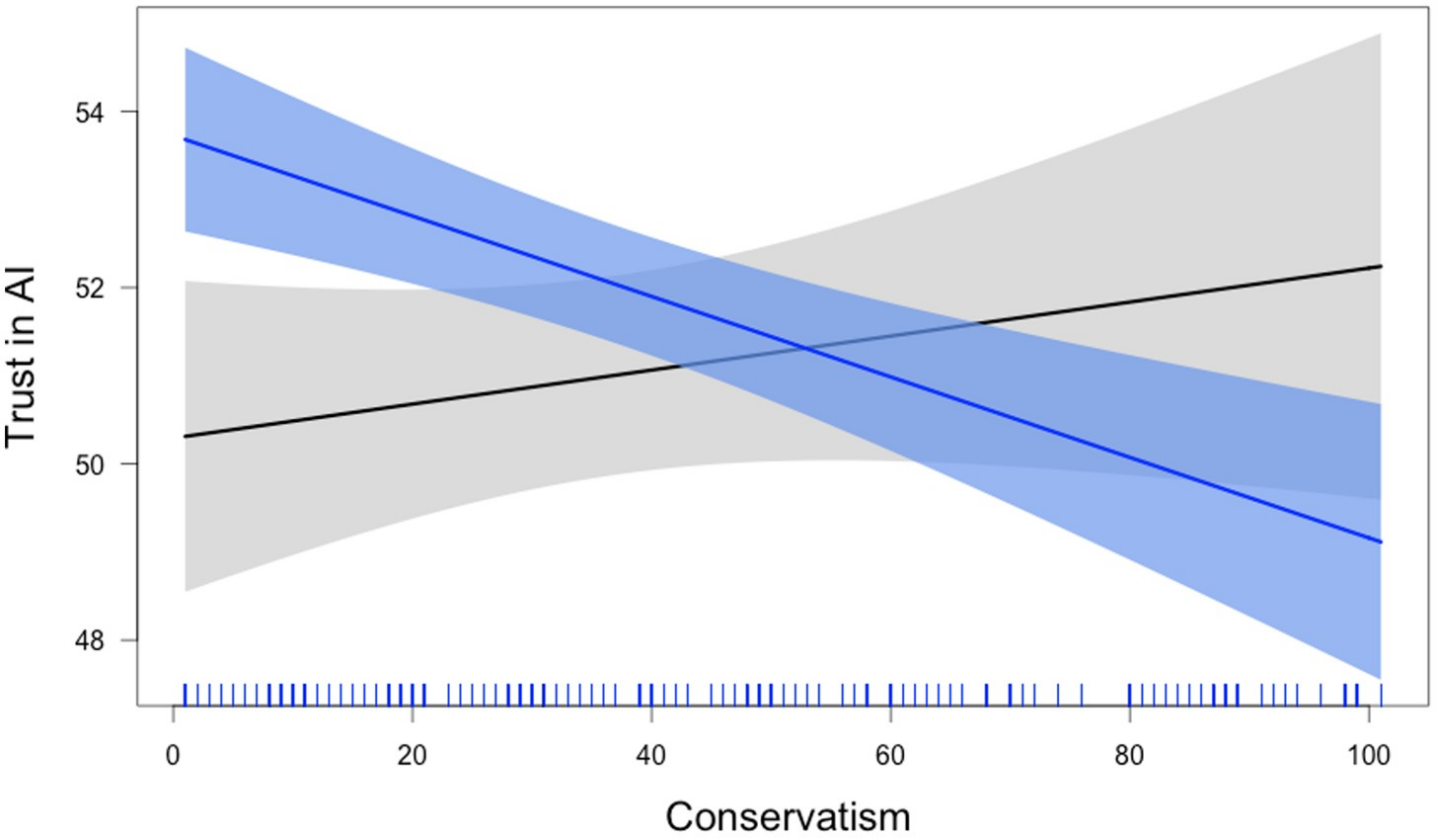
The effect of conservatism on trust in AI is significant for consequential but not inconsequential tasks.

Breaking down the interaction, we found that conservatism predicted trust in AI for consequential tasks, standardized β = -.04, *p* < .001, but not for inconsequential tasks, β = -.01, *p* = .310. Power analysis revealed that we have 82% power to detect the effect size of -.04, and that a sample size of 392 would be required to detect this effect with 80% power. Fig 3 below displays this interaction visually.

### Discussion

Study 2 shows that the effect of conservatism on trust in AI is moderated by the consequentialness of the application for which AI is being considered. For consequential tasks, increasing conservatism predicts decreasing trust in (or increasing aversion to) AI; for inconsequential tasks, conservatism has no effect on trust.

Importantly, the potential of AI to improve outcomes is likely to be the greatest for more consequential tasks. For example, replacing human-driven cars with driverless cars has the potential to save millions of lives that are lost every year to accidents caused by human error, fatigue, alcohol, and so on. At the same time, these same consequential tasks face the greatest resistance to AI among conservatives, posing a major challenge for realizing the potential benefits that AI can offer.

## Study 3

This study is focused on testing the full predicted pattern of moderated mediation: conservatism predicts trust in AI because it predicts the perceived riskiness of AI; because perceived risks are lower for inconsequential tasks, the divide between liberals and conservatives is stronger for tasks that have serious consequences. Study 1 provided mediation evidence for this process, while Study 2 provided moderation evidence. Study 3 combines mediation and moderation to test the entire proposed conceptual model simultaneously.

### Method

Our sample for Study 3 consisted of 550 MTurk users (46% female, mean age = 34.7). We excluded 48 participants from the analysis because they failed a basic attention check, for a final sample of 502. Previous studies forced participants to answer this question correctly as an instructional manipulation check [33], but in this study participants could proceed without answering correctly. The results of this study are unchanged if we include these participants. Note that no attention check was used in Studies 1 and 2.

Participants were randomly assigned to evaluate AI either for purposes of music recommendation (a task with relatively low consequences) or for use in a self-driving car (a task with relatively high consequences). We selected these two tasks because driving a car was rated as a highly consequential task in Study 2 (M = 25.6 on a scale from -50 to 50), whereas recommending music was rated as a highly inconsequential task (M = -18.2). Both conditions began by having participants read the same brief introduction to AI that was used in Study 2 (without mentioning any potential risks associated with AI; see appendix for full stimuli). In the “music” condition, participants then reported how risky it seems and how much they would trust AI to recommend music. In the “car” condition, participants reported how risky it seems and how much they would trust AI to control a driverless car. All measures were on 0–100 scales.

### Results

We tested for moderated mediation using Model 8 in the PROCESS macro for SPSS (Hayes 2013). We specified participants’ social conservatism as the independent variable, perceived riskiness of using AI as the mediator, trust in AI as the dependent variable, and condition (music vs. car) as the moderator. We also included age, gender, income, and education as covariates in the model (see web appendix for the analysis without covariates, and also with fiscal and general conservatism instead of social conservatism. Conservatism, trust, and risk are standardized variables. These analyses are consistent with those reported here). Breaking down the model, we first focus on perceived risk, the proposed mediator. Perceived risk was predicted by task, such that it was higher for AI driving cars (M = 63.72) than for AI recommending music (M = 21.06, *t*(501) = 17.28, *p* < .001). Conservatism also predicted perceived risk (β = .50, *p* < .001). Furthermore, the interaction between task and conservatism was also significant (β = -.23, *p* = .009). Conservatism predicted perceived risk of AI driving a car, such that higher conservatism was associated with higher perceived risk (β = .27, *p* < .001), but conservatism had no effect on the perceived risk of AI recommending music (β = .04, *p* = .480).

We then focus on trust in AI, the dependent variable. Trust was predicted by perceived risk (β = -.61, *p* < .001), but not by task (β < .001, *SE* = .07, *p* = .991), conservatism (β = .17, *p* = .132), nor by the interaction between conservatism and task (β = -.12, *p* = .093). Because perceived risk is a function of conservatism (for highly consequential tasks), these results indicate that conservatism predicts perceived risk (for highly consequential tasks), which in turn predicts trust. Indeed, the indirect effect of conservatism on trust, mediated by risk, was significant in the car condition (B = .23, *SE* = .05, 95% CI = .099 to .247), but not in the music condition (B = .01, *SE* = .02, 95% CI = -.043 to .110). Finally, the index of moderated mediation was significant (B = -.14, 95% CI = -.243 to -.037).

Power analysis revealed that we had >99% power to detect an effect size of .22 and that a sample size of 36 would be required to have 80% power to detect this effect size.

### Discussion

The results thus far have demonstrated that conservatism predicts perceived risk, which in turn predicts trust in AI. Consistent with prior research on the determinants of perceived risk, we have found that perceived risk is partially determined by consequentialness, and shown that conservatism only predicts trust in AI when the task for which AI is being used has significant consequences. However, a problem remains. The tasks for which AI has the greatest potential to meaningfully improve outcomes for individuals and society as a whole tend to have the greatest potential consequences: reducing traffic fatalities by eliminating human error from car accidents, quickly and accurately diagnosing complex diseases and providing appropriate recommendations for treatment, and so on. Thus, the tasks for which AI can provide the most potential benefit are also the tasks for which there exists the greatest resistance (among conservatives) to using AI.

So what, if anything, can be done to reduce this resistance and thereby increase the likelihood that AI can achieve its full potential? We explore this question in Study 4.

## Study 4

Relative to liberals, conservatives are known to interpret ambiguous stimuli as more threatening [34,35] and even have larger amygdalae suggesting a biological basis for more sensitive risk and threat perception [36]. Trying to explicitly convince conservatives to not see AI as risky therefore seems unlikely to succeed. However, conservatives tend to rely on different moral foundations than liberals, often basing their judgments on principles of loyalty, authority, and purity, while liberals tend to emphasize principles of fairness and care [37]. Researchers have discovered that framing arguments in terms of conservative moral foundations can make those arguments much more effective at persuading conservatives, relative to framing arguments in terms of liberal moral foundations [31]. For example, conservatives are more likely to support universal health insurance in the United States if the arguments in favor are based on the moral principle of purity (i.e., reducing the number of infected, diseased people) than if they are based on the moral principle of fairness (i.e., everyone deserves to be healthy) [30].

In this study, we test whether this approach could also be effective at increasing conservative support for AI.

### Method

We recruited 400 American participants from Prolific Academic (42% female, mean age = 26). Participants read one of two arguments in favor of using AI in healthcare, based either on the idea that doing so will reduce the number of diseased and infected people, or on the idea that doing so will ensure more equitable access to quality healthcare (see Web Appendix for full arguments). We then measured support for using AI in healthcare using three items (i.e., “I am in favor of using AI more often in healthcare,” α = .85), plus perceived risk of using AI in healthcare and trust AI used in healthcare, and finally the three dimensions of conservatism (social, fiscal, and general).

### Results

Because the manipulation required reading a 200-word article, we first excluded 32 participants who completed the entire survey in less than 1 minute. We then tested whether any of the three dimensions of conservatism interacted with argument condition in predicting support for healthcare AI. We only found a significant general conservatism × condition interaction (β = .20, *p* = .043), a main effect of conservatism on support (β = -.27, *p* < .001), and a main effect of condition, such that the purity condition was less persuasive (β = -.32, *SE* = .11, *p* = .001). Neither social nor fiscal conservatism interacted with argument condition.

**Fig 4:**
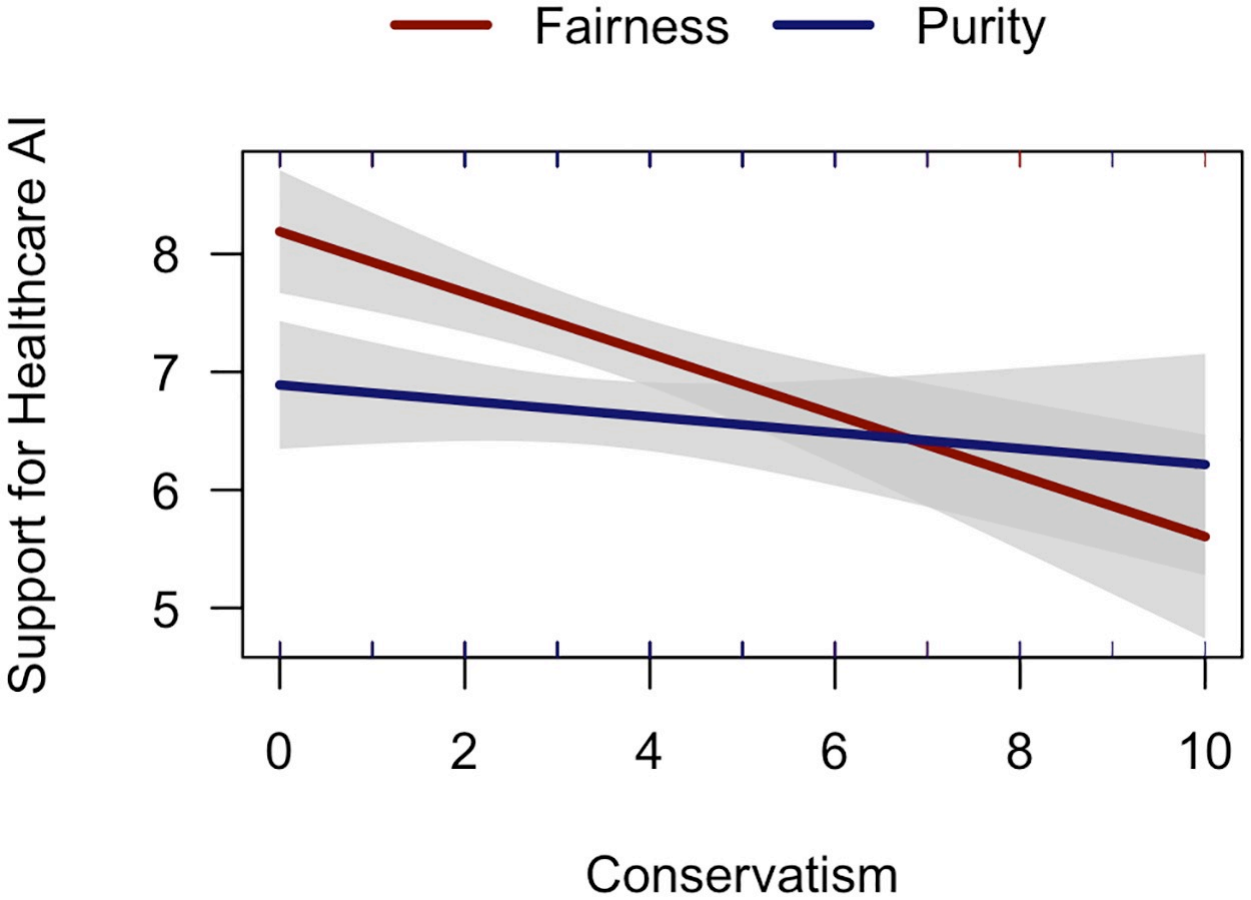
Fairness-based arguments persuade liberals more than purity-based arguments.

Results in the fairness condition were consistent with those from Studies 1–3: conservatism predicted lower support for healthcare AI (standardized β = .27, *p* < .001). In the purity condition, however, conservatism did not predict support (standardized β = .07, *p* = .361; see Fig 4). Power analysis revealed that we have 99% power to detect an effect size of .27 and would need a sample size of 29 to detect this effect size with 80% power. Critically, these diverging patterns did not result from conservatives (defined as being below the scale midpoint) being more persuaded by purity arguments (M = 6.76) than by fairness arguments (M = 6.51, *t*(52) = .47, *p* = .641). Rather, these patterns reflect reduced support among liberals (above the scale midpoint) shown purity arguments (M = 7.07) versus fairness arguments (M = 7.90, *t*(248) = 3.67, *p* < .001).

Social and fiscal conservatism did not interact with condition to predict support, and none of the three dimensions of conservatism interacted with condition to predict perceived riskiness of or trust in healthcare AI.

We were therefore unable to shift conservatives’ attitudes towards a consequential form of AI using a moral reframing approach. Our final study attempts once more to achieve this goal using a revised moral reframing manipulation.

## Study 5

The previous study found that emphasizing different moral foundations shifted liberals’ support for AI but did not change conservatives’ attitudes. In Study 5, we therefore focus specifically on attempting to shift conservatives’ attitudes. Specifically, we attempt to increase the persuasiveness of the moral reframing approach by appealing to three typically conservative moral foundations as opposed to just one as we did in Study 4.

### Method

Prolific Academic pre-screens participants on a variety of factors including political ideology, asking them to self-identify as “liberal,” “conservative,” or “moderate.” We therefore recruited 200 self-identified conservatives (48% female, mean age = 39) and asked them to read one of two ~150-word texts arguing in favor of using AI in healthcare. The conservative text was based on principles of purity (AI will reduce the number of diseased Americans), tradition (technological innovation is the American way), and loyalty (it’s a patriotic duty to ensure all Americans are healthy), while the liberal text was based on principles of fairness and care (AI will ensure more equitable access to high quality healthcare; see Web Appendix for full text). Participants then completed the same measure of support for healthcare in AI used in Study 4, plus the risk and trust measures used throughout this paper.

### Results

We first excluded 12 participants who completed the entire study in less than 40 seconds (the cutoff used in this study was shorter because the manipulation word count was approximately 150 words in this study compared to 200 words in the previous study). Among the remaining participants—all self-identified conservatives—condition had no effect on support for healthcare AI (M_conservative_ = 6.23, M_liberal_ = 6.26, *t*(186) = .11, *p* = .914, Cohen’s *d* = .01). Condition also had no effect on the perceived risk of healthcare AI nor on trust in its use (*p*’s > .204).

It therefore appears that framing a consequential application of AI in terms of conservative moral foundations may not be effective at reducing conservative aversion to such uses of AI.

## General discussion

Using artificial intelligence to automate complex tasks has the potential to radically improve outcomes in a fast-growing number of domains, from reducing traffic accidents caused by human error to diagnosing diseases with greater speed and accuracy. The extent to which these improved outcomes are realized, however, depends to a large degree on people’s willingness to trust and rely on AI rather than on themselves or other human beings for performing the task at hand. Understanding when and why people are willing to trust AI is therefore an important task, especially if that understanding can shed light on how to increase trust in AI when high trust is justified by superior performance. This paper begins to make progress in that direction by highlighting the role of a prominent psychological variable–conservatism—in shaping trust in AI. In addition to uncovering the basic relationship between conservatism and trust, we also provide evidence of the psychological process underlying this relationship and show that it is resistant to a moral reframing intervention intended to reduce the effect.

Our analyses cast doubt on two alternative explanations of our results. First, conservatism is often correlated with income, such that our results could be driven by poorer individuals whose jobs are more immediately at risk of being automated by AI, and who happen to also be more conservative. Second, conservatism is often correlated with level of education, such that our results could be driven by less educated individuals who are less knowledgeable about technology and/or more likely to work in jobs that are at immediate risk of automation. These explanations become less likely in light of the fact that the effect of social conservatism on the perceived riskiness of (and trust in) AI remains highly significant even after controlling for participants’ income and level of education, and the fact that these other demographic variables do not themselves have any effect on perceived risk and trust.

Our results contribute to a growing literature on the changing relationship between humans and technology. As technology becomes increasingly capable of automating consequential tasks, research has found that humans are reluctant to trust and rely on technology for tasks traditionally performed by humans [8,10,12]. Our research has uncovered conservatism as an important driver of this aversion to AI.

Political or ideological sources of AI aversion are particularly important given the inextricable links between politics and the impacts that AI has on society. The relationship between conservatism and AI aversion that we have focused on has political implications. For example, it seems likely that both conservative individuals as well as conservative state governments will be slower to adopt AI technologies compared to their liberal counterparts, potentially depriving conservatives of the benefits that AI can provide when it outperforms humans. Similarly, ideologically divided legislative bodies may be unable to reach consensus on regulating AI in nationally important domains.

Future research can continue exploring factors that contribute to AI aversion. For example, prior research has found that people who feel low in power also feel a lower degree of control over anthropomorphized objects [38], which suggests that feelings of power and control are likely to be relevant factors in shaping trust in AI. Indeed, giving people the ability to slightly modify the output of an algorithm has been found to increase the use of that algorithm, further pointing to the importance of perceived control over technology [15]. In light of the previously discussed relationships between conservatism, education, and income, which increase the likelihood that conservative individuals work in jobs that are at high risk of automation, these findings suggest that perceived control over automation technologies such as AI may also contribute to our observed relationship between conservatism and distrust in AI.

Another factor that may be worth exploring in more detail is gender. Females perceived more risk in AI and trusted AI less than males in all of our studies. Females are known to perceive more risk in general [39] and to take fewer risks than men [40]. This may be rooted in evolutionary pressures: whereas males were traditionally hunters who needed to be risk-seeking in order to provide food for their families, females were traditionally child care providers who needed to avoid harm [41,42]. Consistent with an account of gender differences rooted in risk perception, we also found that gender predicted perceived risks of and trust in AI for consequential tasks but not for consequential tasks. Since consequential tasks are less risky, this suggests that females may trust AI less than men because they perceive AI as more risky, but that this gender difference is eliminated for inherently low-risk tasks. However, none of the interventions that we tested in Study 4 interacted with gender. This highlights the need for future research to test different interventions that are specifically rooted in a deeper understanding of gender differences.

Our findings in Studies 4 and 5 suggest that moral reframing interventions may not be universally successful across topic domains. Given that this approach has been successful in increasing conservative support for typically liberal causes such as the Affordable Care Act and environmentally sustainable behaviors [35,43], it is perhaps surprising that it failed to shift conservative attitudes in this context. By opening our file drawer and reporting these null results, we hope to encourage further research on when and why moral reframing is most effective at mitigating ideological divides in attitudes. For example, it is possible that the kind of reframing interventions we tested would be more effective if they were delivered by trusted conservative sources rather than by university researchers in an online survey.

Relatedly, an important limitation of this research concerns the specific context in which AI is developed and marketed. Specifically, the primary developers and marketers of AI are relatively liberal corporations such as Google and Facebook, which are often criticized for being hostile to conservative viewpoints. Our results cannot definitively rule out the notion that conservatives are averse to AI because they are averse to the companies that produce AI, rather than being averse to AI in particular for reasons unrelated to its developers.

We also acknowledge three further limitations of our research. First, we did not provide a great level of detail to participants regarding the specific tasks that AI performs and how those tasks are shared with humans. For example, AI in medical contexts may be used in collaboration with a human doctor, and attitudes may be different in this context compared to if AI is used alone without human collaboration. Second, while we controlled for a range of demographic factors in our analyses, we did not measure some that may be relevant, including participants’ occupation or specific field of training. Finally, the amount of variance that our regression models explain is relatively modest, ranging from about 5–10%. While these numbers are typical for psychological research, we must acknowledge that comfort with and perceived risks of AI are of course complex variables with many different inputs and influences. The effects of conservatism that we have uncovered are therefore just one relatively small influence on our dependent variables of interest.

As AI research continues to progress, the potential benefits that this technology can offer continue to grow. Increasing trust in AI can therefore increase the probability that these benefits are realized. At the same time, AI also brings novel risks to people and to society, as recognized by many of the participants in our studies. These risks range from mass unemployment and algorithm-entrenched inequality and discrimination, to the loss of skills as tasks are offloaded to AI, and more [44,45]. Any efforts to increase trust in AI should therefore proceed mindfully—not merely to increase trust in all forms of AI at all times, but to ensure that such efforts are focused on applications of AI that are clearly beneficial to society, and whose risks are minimized. This requires the companies developing AI and the governments regulating them to develop policies specifically addressing these and other risks [46,47]. Understanding how people perceive AI can help inform the development of those policies and the development of AI and AI-based products more broadly. The research presented in this paper contributes to such an understanding.

## Supporting information

S1 File (DOCX)
