# Supplementary material for: Conservatism predicts aversion to consequential Artificial Intelligence
Source: PLoS One. 2021 Dec 20;16(12):e0261467. doi: 10.1371/journal.pone.0261467 (PMC8687590; doi:10.1371/journal.pone.0261467)
Supplement: S1 File — (DOCX) [file pone.0261467.s001.docx]

WEB APPENDIX A

This appendix provides the stimuli used in all studies.

*STUDY 1*

In this survey, we are interested in your thoughts on **Artificial Intelligence**.

 ---

Every year, Artificial Intelligence (AI) becomes capable of performing new tasks that only humans could do before, from beating chess grandmasters and the best human players on the game show Jeopardy!, to driving cars and diagnosing diseases.

Ongoing developments in Artificial Intelligence include the creation of systems intended to handle increasingly complex tasks--for example, reading medical journals, diagnosing diseases, delivering treatment recommendations, and even controlling automated driverless cars.

 ---

 We are interested in your thoughts about Artificial Intelligence and its potential use in different areas of society. To begin, please respond to the following questions:

|  |
| --- |

Many people are worried that developing AI is risky for several reasons. For example, AI can already do many jobs better than humans can, and the number of such jobs is growing all the time - including jobs like truck drivers and fast food workers and even some kinds of lawyers and doctors. One risk is therefore that developing AI will put many humans out of a job. 


Another risk that people worry about is that AI could potentially become even more intelligent than humans and start to develop its own goals. This could mean that AI decides humans are no longer helpful for its development and could start to manipulate or hurt humans. 

Given these concerns, how risky do you think it is to develop and rely on AI?

|  | Not at all | Extremely |
| --- | --- | --- |

|  | 0 | 10 | 20 | 30 | 40 | 50 | 60 | 70 | 80 | 90 | 100 |
| --- | --- | --- | --- | --- | --- | --- | --- | --- | --- | --- | --- |

|  | 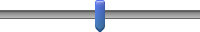 |
| --- | --- |

|  |
| --- |

How comfortable are you with the idea of relying on AI?

|  | Not at all | Extremely |
| --- | --- | --- |

|  | 0 | 10 | 20 | 30 | 40 | 50 | 60 | 70 | 80 | 90 | 100 |
| --- | --- | --- | --- | --- | --- | --- | --- | --- | --- | --- | --- |

|  | 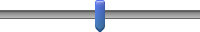 |
| --- | --- |

|  |
| --- |

How risky do you think it is to rely on AI for medical tasks such as diagnosing diseases and providing treatment recommendations? In this context, the potential risks involve the AI mis-diagnosing a disease (either missing a disease that is there, or thinking one disease is actually a different disease), or the AI could provide incorrect or harmful treatment recommendations.

|  | Not at all | Extremely |
| --- | --- | --- |

|  | 0 | 10 | 20 | 30 | 40 | 50 | 60 | 70 | 80 | 90 | 100 |
| --- | --- | --- | --- | --- | --- | --- | --- | --- | --- | --- | --- |

|  | 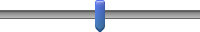 |
| --- | --- |

|  |
| --- |

How risky do you think it is to rely on AI for controlling driverless cars? In this context, the potential risks involve the AI cause an accident that injures or even kills the passengers in the car or in other cars, or pedestrians.

|  | Not at all | Extremely |
| --- | --- | --- |

|  | 0 | 10 | 20 | 30 | 40 | 50 | 60 | 70 | 80 | 90 | 100 |
| --- | --- | --- | --- | --- | --- | --- | --- | --- | --- | --- | --- |

|  | 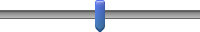 |
| --- | --- |

How comfortable are you with the idea of relying on AI for medical tasks such as diagnosing diseases and providing treatment recommendations?

|  | Not at all | Extremely |
| --- | --- | --- |

|  | 0 | 10 | 20 | 30 | 40 | 50 | 60 | 70 | 80 | 90 | 100 |
| --- | --- | --- | --- | --- | --- | --- | --- | --- | --- | --- | --- |

|  | 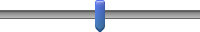 |
| --- | --- |

|  |
| --- |

How comfortable are you with the idea of relying on AI for controlling driverless cars?

|  | Not at all | Extremely |
| --- | --- | --- |

|  | 0 | 10 | 20 | 30 | 40 | 50 | 60 | 70 | 80 | 90 | 100 |
| --- | --- | --- | --- | --- | --- | --- | --- | --- | --- | --- | --- |

|  | 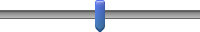 |
| --- | --- |

|  |
| --- |

*DEMOGRAPHIC QUESTIONS (INCLUDED IN ALL STUDIES):*

How old are you?

________________________________________________________________

What gender are you?

Male (1)

Female (2)

What is your household income?

$0 to $14,9999 (1)

$15,000 to $24,999 (2)

$25,000 to $34,999 (3)

$35,000 to $49,999 (4)

$50,000 to $74,999 (5)

$75,000 to $99,999 (6)

$100,000 and over (7)

|  |
| --- |

What is the highest level of education you have completed?

Less than High School (1)

High School / GED (2)

Some College (3)

2-year College Degree (4)

4-year College Degree (5)

Graduate Degree (6)

|  |  |
| --- | --- |

How conservative or liberal do you feel about:

|  | Very conservative | Moderate | Very liberal |
| --- | --- | --- | --- |

|  | 0 | 10 | 20 | 30 | 40 | 50 | 60 | 70 | 80 | 90 | 100 |
| --- | --- | --- | --- | --- | --- | --- | --- | --- | --- | --- | --- |

| In general (3) | 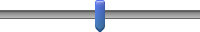 |
| --- | --- |
| Social issues (1) | 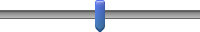 |
| Financial issues (2) | 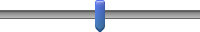 |

*STUDY 2*

*PART 1 (task consequentialness ratings)*

Please use the sliders to indicate how much each of the tasks below seems to be objective vs. subjective. 

Objective means based in facts that are quantifiable and measurable. 

Subjective means open to interpretation and based in personal opinion.

|  | Completely subjective | Completely objective |
| --- | --- | --- |

*Participants rated each of the 27 tasks shown in Figure 3 in the main text.*

*PART 2 (trust in AI ratings)*

Artificial intelligence (AI) is a set of computer programs that can be used to accomplish a task. Thanks to rapid progress in computer science, AI can now be used to accomplish a wide range of tasks. 


Please use the sliders to indicate how much you would trust AI to perform each of the tasks below.

|  | Not trust   AI at all | Trust    AI completely |
| --- | --- | --- |

|  | 0 | 10 | 20 | 30 | 40 | 50 | 60 | 70 | 80 | 90 | 100 |
| --- | --- | --- | --- | --- | --- | --- | --- | --- | --- | --- | --- |

*Participants rated each of the 27 tasks shown in Figure 3 in the main text.*

*STUDY 3*

Artificial intelligence (AI) refers to a set of computer programs that can be used to accomplish a task. Thanks to rapid progress in computer science, AI can now be used to accomplish a wide range of tasks that humans would generally do, without being explicitly instructed how to do so by humans.

*MUSIC CONDITION*

How much would you trust Artificial Intelligence to provide music recommendations?

|  | Not at all | Extremely |
| --- | --- | --- |

|  | 0 | 10 | 20 | 30 | 40 | 50 | 60 | 70 | 80 | 90 | 100 |
| --- | --- | --- | --- | --- | --- | --- | --- | --- | --- | --- | --- |

|  | 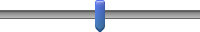 |
| --- | --- |

In your opinion, how risky does it seem to use Artificial Intelligence to provide music recommendations?

|  | Not at all | Extremely |
| --- | --- | --- |

|  | 0 | 10 | 20 | 30 | 40 | 50 | 60 | 70 | 80 | 90 | 100 |
| --- | --- | --- | --- | --- | --- | --- | --- | --- | --- | --- | --- |

|  | 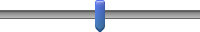 |
| --- | --- |

|  |
| --- |

*CAR CONDITION*

How much would you trust Artificial Intelligence to drive a car?

|  | Not at all | Extremely |
| --- | --- | --- |

|  | 0 | 10 | 20 | 30 | 40 | 50 | 60 | 70 | 80 | 90 | 100 |
| --- | --- | --- | --- | --- | --- | --- | --- | --- | --- | --- | --- |

|  | 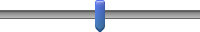 |
| --- | --- |

In your opinion, how risky does it seem to use Artificial Intelligence to drive a car?

|  | Not at all | Extremely |
| --- | --- | --- |

|  | 0 | 10 | 20 | 30 | 40 | 50 | 60 | 70 | 80 | 90 | 100 |
| --- | --- | --- | --- | --- | --- | --- | --- | --- | --- | --- | --- |

|  | 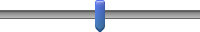 |
| --- | --- |

*STUDY 4*

*PURITY CONDITION*

Human errors by healthcare providers practically ensures that we will have unclean, infected, and diseased Americans walking among us. Deploying artificial intelligence (AI) to provide more healthcare services can help.

Many diseases, such as COVID-19, tuberculosis, hepatitis, toxocariasis, and other viruses or parasites can easily spread throughout the population. These diseases are disgusting infestations that invade the human body and leech out needed nutrients to survive. Many of these diseases have grotesque symptoms like yellowing of the skin and eyes, coughing up bloody mucus, itchy rashes, and lesions. These diseases are contagious and spread through the population infecting many.

The spread of these diseases, however, could be greatly reduced if we used AI more often in healthcare. Human doctors make so many mistakes in diagnosis, prognosis, and treatment decisions that 250,000 Americans die every year because of human errors in healthcare. This makes human error in medicine the third leading cause of death after cancer and health disease.

If we used AI instead of human doctors, we would eliminate human error from healthcare. Individuals infected with these contagious diseases would become much less likely to spread their sickness to others, because AI would be able to detect and treat them with fewer errors.

Using AI in healthcare represents a major step towards the extermination of infectious diseases. Medical AI is a way of purifying America from some of its most infectious diseases, making it less and less likely that healthy individuals will ever encounter these diseases. Everyone should have healthcare, and AI can make it happen.

*FAIRNESS CONDITION*

In its current state healthcare in the U.S. is inherently unfair and unjust. Deploying artificial intelligence (AI) to provide more healthcare services can ensure that everyone, not just the rich and the fortunate, has access to the best care available.

The quality of healthcare in America is unfairly dependent on your wealth. Many can’t afford the best care or the most comprehensive insurance, so they are unfairly denied access to resources their wealthier countrymen receive, simply because they make less money.

Healthcare for everyone would be improved if we used AI more often in healthcare. Human doctors make so many mistakes in diagnosis, prognosis, and treatment decisions that 250,000 Americans die every year because of human errors in healthcare. This makes human error in medicine the third leading cause of death after cancer and health disease.

If we used AI instead of human doctors, we would eliminate human error and care would also be more standardized so that everyone gets the same quality of care regardless of their income. No more rich people getting access to the best human doctors while the poor are stuck with low-quality care – with AI, the best quality care could be available through every computer in every hospital in the country.

It all comes down to simple questions: Shouldn’t all people equally have the right to the best, error-free care? Is it fair to allow people to suffer from poor treatment, simply because they are poor? Is this justice for all? Access to quality healthcare is a basic right not a luxury reserved for the rich. Everyone should have healthcare, and AI can make it happen.

After reading this, how much do you agree with these statements?

I am in favor of using AI more often in healthcare.

|  | Strongly disagree | Strongly agree |
| --- | --- | --- |

|  | 0 | 1 | 2 | 3 | 4 | 5 | 6 | 7 | 8 | 9 | 10 |
| --- | --- | --- | --- | --- | --- | --- | --- | --- | --- | --- | --- |

|  | 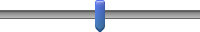 |
| --- | --- |

There is no need to use AI more often in healthcare.

|  | Strongly disagree | Strongly agree |
| --- | --- | --- |

|  | 0 | 1 | 2 | 3 | 4 | 5 | 6 | 7 | 8 | 9 | 10 |
| --- | --- | --- | --- | --- | --- | --- | --- | --- | --- | --- | --- |

|  | 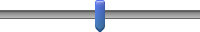 |
| --- | --- |

Using AI in healthcare can solve many of America's healthcare problems.

|  | Strongly disagree | Strongly agree |
| --- | --- | --- |

|  | 0 | 1 | 2 | 3 | 4 | 5 | 6 | 7 | 8 | 9 | 10 |
| --- | --- | --- | --- | --- | --- | --- | --- | --- | --- | --- | --- |

|  | 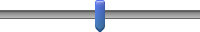 |
| --- | --- |

In your opinion, how risky does it seem to use AI in healthcare?

|  | Not at all | Extremely |
| --- | --- | --- |

|  | 0 | 1 | 2 | 3 | 4 | 5 | 6 | 7 | 8 | 9 | 10 |
| --- | --- | --- | --- | --- | --- | --- | --- | --- | --- | --- | --- |

|  | 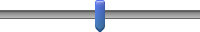 |
| --- | --- |

trust
How much would you trust AI being used in healthcare?

|  | Not at all | Extremely |
| --- | --- | --- |

|  | 0 | 1 | 2 | 3 | 4 | 5 | 6 | 7 | 8 | 9 | 10 |
| --- | --- | --- | --- | --- | --- | --- | --- | --- | --- | --- | --- |

How conservative or liberal do you feel about:

|  | Very conservative | Moderate | Very liberal |
| --- | --- | --- | --- |

|  | 0 | 1 | 2 | 3 | 4 | 5 | 6 | 7 | 8 | 9 | 10 |
| --- | --- | --- | --- | --- | --- | --- | --- | --- | --- | --- | --- |

| In general () | 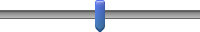 |
| --- | --- |
| Social issues () | 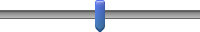 |
| Financial issues () | 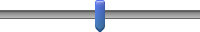 |

*STUDY 5*

*CONSERVATIVE FOUNDATIONS CONDITION*

250,000 Americans die every year from human error in healthcare. Using artificial intelligence (AI) in healthcare can help. We should do this for 3 reasons.

1. **AI will reduce the number of infected and diseased Americans.** AI can diagnose diseases more accurately than human doctors, so diseases can be caught and treated earlier. More AI means less human error and less disgusting disease.
2. **High-tech innovation is the American way of life.** This country was built on innovations like Edison’s light bulb and Franklin’s eyeglasses. Some people will always criticize innovation and change, but you have to admit it’s the American way.
3. **It’s patriotic to ensure the health of all Americans**. Our citizens’ health is our nation’s greatest resource. It’s our patriotic duty to make sure we’re all as healthy as we can be, and AI can get us there with its superior diagnosis and treatment recommendations.

*LIBERAL FOUNDATIONS CONDITION*

250,000 Americans die every year from human error in healthcare. Using artificial intelligence (AI) in healthcare can help. We should do this for 3 reasons.

1. **AI lets us provide better care for the needy.** AI can diagnose diseases more accurately than human doctors and, so diseases can be caught and treated earlier. More AI means less human error and more compassionate harm reduction.
2. **The current healthcare system is unfair for the poor.** Today, the rich can buy access to much higher quality healthcare than the poor. Human error is more likely to harm the poor. Quality healthcare should be a right for everyone.
3. **AI will standardize healthcare so the rich and poor get the same quality.** Unlike human doctors, AI is standardized and scalable: once we have it, we can use it in every hospital in the country. Everyone can get the same benefits from AI, regardless of income.

All measures identical to Study 4.

WEB APPENDIX B

This appendix provides the details of additional analyses not reported in the main text.

**STUDY 1**

In the main text, we report how *social* conservatism affects the perceived risk of AI and participants’ comfort relying on AI for different tasks, and in general. Below, we provide the results of the same analyses using *fiscal* conservatism and *conservatism in general.*

Fiscal Conservatism

**Table S1: Fiscal Conservatism-Only and Fiscal Conservatism + Demographics Models of Comfort with AI, Study 1**

| Model | Conservatism Only 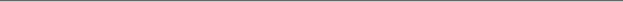 | | | Conservatism + Demographics 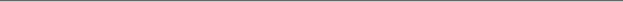 | | | |
| --- | --- | --- | --- | --- | --- | --- | --- |
| Domain | Medical Diagnoses | Self -Driving Cars | General | | Medical Diagnoses | Self-  Driving  Cars | General |
| 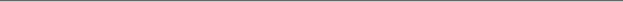 |  |  | 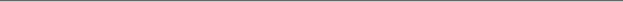 |  | |  |  |
| Fiscal Conservatism | -.13 (.05)* | -.18 (.05)** | -.13 (.04)** | -.15 (.05)** | | -.18 (.05)** | -.14 (.05)** |
| Education: |  |  |  |  | |  |  |
| High school |  |  |  | | 17.56 (17.04) | 29.85 (17.55)† | 35.40 (15.20)* |
| Some college |  |  |  | 12.50 (16.75) | | 32.14 (17.24)† | 31.15 (14.93)* |
| 2-year college |  |  |  | 18.32 (16.99) | | 36.80 (17.50)* | 39.34 (15.15)* |
| 4-year college |  |  |  | 15.79 (16.69) | | 33.64 (17.85)† | 37.01 (14.88)* |
| Graduate degree |  |  |  | 19.23 (17.15) | | 36.16 (17.66)* | 39.20 (15.30)* |
| Income |  |  |  | .04 (.91) | | 0.64 (.94) | -0.53 (.81) |
| Age |  |  |  | .08 (.14) | | -.23 (.14)† | 0.06 (.12) |
| Female |  |  |  | -10.54 (2.97)** | | -11.30 (3.06)** | -10.57 (2.65)** |
| Intercept | 40.55 (3.10)* | 36.32 (3.23)** | 45.15 (2.79)** | 34.99 (18.08)† | | 24.90 (18.62) | 23.85 (16.12) |
| *R^2^* | .02 | .03 | .02 | .05 | | .09 | .07 |

Gender (“Female”) is dummy coded with “Male” as the reference group.

Education variables are dummy coded with “less than high school” as the reference group.

† = *p* < .10 * = *p* < .05 ** = *p* < .01

**Table S2: Fiscal Conservatism-Only and Fiscal Conservatism + Demographics Models of Perceived Risks of AI, Study 1**

| Model | Conservatism Only 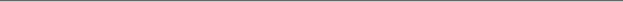 | | | Conservatism + Demographics 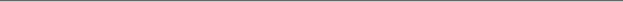 | | | |
| --- | --- | --- | --- | --- | --- | --- | --- |
| Domain | Medical Diagnoses | Self -Driving Cars | General | | Medical Diagnoses | Self-  Driving  Cars | General |
| 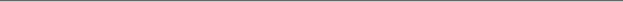 |  |  | 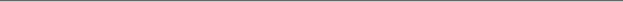 |  | |  |  |
| Fiscal Conservatism | -.05 (.05) | -.21 (.05)** | .00 (.04) | -.07 (.05) | | -.13 (.05)** | -.03 (.05) |
| Education: |  |  |  |  | |  |  |
| High school |  |  |  | | -13.67 (15.62) | -23.02 (16.15) | -25.87 (15.01)* |
| Some college |  |  |  | -18.61 (15.35) | | -32.56 (15.87)* | -29.80 (14.75)* |
| 2-year college |  |  |  | -17.39 (15.59) | | -34.79 (16.10)* | -32.01 (14.96)* |
| 4-year college |  |  |  | -17.62 (15.30) | | -30.72 (15.82)† | -28.05 (14.70)* |
| Graduate degree |  |  |  | -19.45 (15.73) | | -37.00 (16.26)* | -27.71 (15.10)† |
| Income |  |  |  | -0.02 (.83) | | -.27 (.86) | -0.72 (.80) |
| Age |  |  |  | -0.08 (.13) | | .15 (.14) | -0.17 (.12) |
| Female |  |  |  | 10.97 (2.73)** | | 10.12 (2.82)** | 7.20 (2.62)** |
| Intercept | 62.46 (2.86)* | 66.24 (2.98)** | 59.22 (2.73)* | 68.04 (16.58)** | | 78.32 (17.35)** | 88.33 (15.92)** |
| *R^2^* | .01 | .04 | .00 | .05 | | .08 | .04 |

Gender (“Female”) is dummy coded with “Male” as the reference group.

Education variables are dummy coded with “less than high school” as the reference group.

† = *p* < .10 * = *p* < .05 ** = *p* < .01

General Conservatism

**Table S3: General Conservatism-Only and General Conservatism + Demographics Models of Comfort with AI, Study 1**

| Model | Conservatism Only 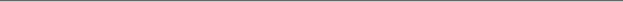 | | | Conservatism + Demographics 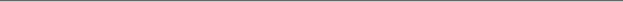 | | | |
| --- | --- | --- | --- | --- | --- | --- | --- |
| Domain | Medical Diagnoses | Self -Driving Cars | General | | Medical Diagnoses | Self-  Driving  Cars | General |
| 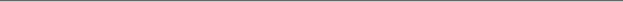 |  |  | 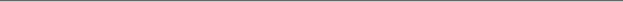 |  | |  |  |
| General Conservatism | -.12 (.05)* | -.20 (.05)** | -.12 (.04)** | -.14 (.05)* | | -.20 (.05)** | -.13 (.05)** |
| Education: |  |  |  |  | |  |  |
| High school |  |  |  | | 35.14 (15.23) | 29.25(17.54)† | 35.14 (15.23)* |
| Some college |  |  |  | 31.80 (14.97)* | | 31.40(17.23)† | 31.24 (14.95)* |
| 2-year college |  |  |  | 38.80 (15.18)* | | 31.80 (14.97)* | 38.80 (15.18)* |
| 4-year college |  |  |  | 36.71 (14.92)* | | 32.89 (17.18)† | 36.71 (14.92)* |
| Graduate degree |  |  |  | 38.61 (15.35)* | | 34.78(17.67)* | 38.61 (15.35)* |
| Income |  |  |  | .52 (.91) | | .71 (.93) | -0.52 (.81) |
| Age |  |  |  | .04 (.12) | | -0.24(.14)† | 0.04 (.12) |
| Female |  |  |  | -10.10 (2.97)** | | -11.00 (3.04)** | -10.25 (2.64)** |
| Intercept | 40.43 (3.43)* | 34.52 (3.56)** | 44.47 (3.09)* | 36.24 (18.16)* | | 24.15 (18.62) | 23.37 (16.17) |
| *R^2^* | .01 | .03 | .02 | .05 | | .09 | .07 |

Gender (“Female”) is dummy coded with “Male” as the reference group.

Education variables are dummy coded with “less than high school” as the reference group.

† = *p* < .10 * = *p* < .05 ** = *p* < .01

**Table S4: General Conservatism-Only and General Conservatism + Demographics Models of Perceived Risks of AI, Study 1**

| Model | Conservatism Only 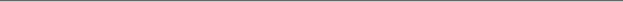 | | | Conservatism + Demographics 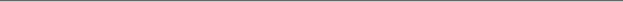 | | | |
| --- | --- | --- | --- | --- | --- | --- | --- |
| Domain | Medical Diagnoses | Self -Driving Cars | General | | Medical Diagnoses | Self-  Driving  Cars | General |
| 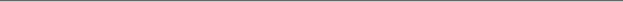 |  |  | 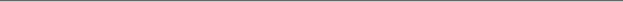 |  | |  |  |
| General Conservatism | -.11 (.05)* | .19 (.05)** | -.02 (.04) | -.13 (.05)* | | -.19 (.05)** | -.05 (.05)** |
| Education: |  |  |  |  | |  |  |
| High school |  |  |  | | -12.81 (15.54) | -22.06 (16.03) | -25.58 (15.00)† |
| Some college |  |  |  | -17.65 (15.27) | | -31.47 (15.75)* | -29.48 (14.74)* |
| 2-year college |  |  |  | -16.67 (15.50) | | -33.82 (15.98)* | -31.73 (14.95)* |
| 4-year college |  |  |  | -16.47 (15.21) | | -29.46 (15.75)† | -27.67 (14.69)† |
| Graduate degree |  |  |  | -17.46 (15.65) | | -34.78 (16.15)* | -27.03 (15.12)* |
| Income |  |  |  | -.18 (.83) | | -0.42 (.85) | -0.78 (.80) |
| Age |  |  |  | .10 (.12) | | 0.14 (.13) | -.18 (.12) |
| Female |  |  |  | 11.21 (2.70)** | | 10.21 (2.78)** | 7.25 (2.61)** |
| Intercept | 66.36 (3.14)* | 70.27 (3.27)** | 60.57 (3.01)* | 71.89 (16.50)** | | 81.91 (17.02)** | 89.50 (15.93) |
| *R^2^* | .01 | .04 | .00 | .05 | | .10 | .04 |

Gender (“Female”) is dummy coded with “Male” as the reference group.

Education variables are dummy coded with “less than high school” as the reference group.

† = *p* < .10 * = *p* < .05 ** = *p* < .01

**STUDY 2**

In the main text, we defined consequential tasks as those rated above the midpoint of the scale used to measure consequentialism, and inconsequential tasks as those rated below the midpoint. This resulted in 21 consequential tasks and 6 inconsequential tasks, with substantial variation of consequentialness within each group of tasks. Here, we report similar analyses using different cutoffs for defining consequential tasks. Importantly, the consequentialness by conservatism replicates in each case.

First, we define consequential tasks as those rated above +10 and inconsequential tasks as those rated as below -10 (on a scale from -50 to + 50).

**Table S5. Social Conservatism-Only and Social Conservatism + Demographics Models of Trust in AI, with task consequentialness defined as +10 / -10, Study 2**

| Model | Conservatism + Consequentialness Only 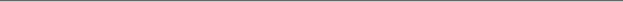 | Conservatism + Consequentialness + Demographics 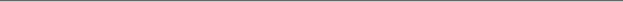 |
| --- | --- | --- |
|  | Trust in AI | Trust in AI |
| Social Conservatism | -.02 (.02) | -.02 (.02) |
| Consequentialness | -8.09 (1.75)** | -8.09 (1.74)** |
| Conservatism * Consequentialness Interaction | .07 (.02)** | .07 (.02)** |
| Participant | .00 (.00) | .00 (.00 |
| Age |  | -.10 (.03)** |
| Female |  | -4.15 (.69)** |
| Intercept | 53.32 (1.46)** | 62.66 (2.11)** |
| *R^2^* | .00 | .01 |

Gender (“Female”) is dummy coded with “Male” as the reference group.

† = *p* < .10 * = *p* < .05 ** = *p* < .01

Next, we define consequential tasks as those rated above +15 and inconsequential tasks as those rated as below -15 (on a scale from -50 to + 50).

**Table S6. Social Conservatism-Only and Social Conservatism + Demographics Models of Trust in AI, with task consequentialness defined as +15 / -15, Study 2.**

| Model | Conservatism + Consequentialness Only 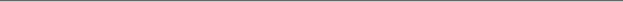 | Conservatism + Consequentialness + Demographics 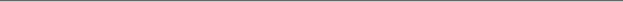 |
| --- | --- | --- |
|  | Trust in AI | Trust in AI |
| Social Conservatism | -.02 (.02) | -.02 (.02) |
| Consequentialness | -5.63 (1.89)** | -5.63 (1.88)** |
| Conservatism * Consequentialness Interaction | .08 (.03)** | .08 (.03)** |
| Participant | .00 (.00) | .00 (.00 |
| Age |  | -.12 (.03)** |
| Female |  | -4.69 (.72)** |
| Intercept | 52.16 (1.60)** | 62.87 (2.28)** |
| *R^2^* | .00 | .01 |

Gender (“Female”) is dummy coded with “Male” as the reference group.

† = *p* < .10 * = *p* < .05 ** = *p* < .01

We also report a similar analysis (with consequentialness defined as +10/-10) but using fiscal conservatism instead of social conservatism. Conservatism in general was not measured in this study.

**Table S7. Fiscal Conservatism-Only and Fiscal Conservatism + Demographics Models of Trust in AI, with task consequentialness defined as +10 / -10, Study 2**

| Model | Conservatism + Consequentialness Only 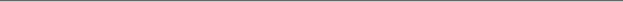 | Conservatism + Consequentialness + Demographics 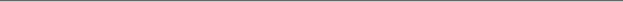 |
| --- | --- | --- |
|  | Trust in AI | Trust in AI |
| Social Conservatism | -.01 (.02) | -.01 (.02) |
| Consequentialness | -5.82 (1.56)** | -5.82 (1.55)** |
| Conservatism * Consequentialness Interaction | .05 (.02)† | .05 (.02)† |
| Participant | .00 (.00) | .00 (.00 |
| Age |  | -.11 (.03)** |
| Female |  | -4.13 (.69)** |
| Intercept | 52.46 (1.42)** | 62.18 (1.97)** |
| *R^2^* | .00 | .00 |

Gender (“Female”) is dummy coded with “Male” as the reference group.

† = *p* < .10 * = *p* < .05 ** = *p* < .01

**STUDY 3**

In the main text, we report the results of the moderated mediation analysis using social conservatism to predict trust in AI, mediated by perceived risk, moderated by task, and including age, gender, education, and income as covariates. As in previous studies, gender predicted trust and risk, such that women perceived more risk and trusted AI less. No other covariates had significant effects on the mediator or dependent variable. In this supplementary analysis, we report the same analysis without covariates, and a similar analysis using fiscal and general conservatism instead.

Without covariates, the results are as follows. We specified participants’ social conservatism as the independent variable, perceived riskiness of using AI as the mediator, trust in AI as the dependent variable, and condition (music vs. car) as the moderator. Breaking down the model, we first focus on perceived risk, the proposed mediator. Perceived risk was predicted by task, such that it was higher for AI driving cars (M = 63.72) than for AI recommending music (M = 21.06, *t*(503) = 17.28, *p* < .001). Conservatism also predicted perceived risk (β = .27, *p* < .001). Furthermore, the interaction between task and conservatism was also significant (β = -.23, *p* = .005). Conservatism predicted perceived risk of AI driving a car, such that higher conservatism was associated with higher perceived risk (β = .27, *p* < .001), but conservatism had no effect on the perceived risk of AI recommending music (β = -.05, *p* = .437).

We then focus on trust in AI, the dependent variable. Trust was predicted by perceived risk (β = -.64, *p* < .001), but not by task (β = 1.59, *p* = .740), conservatism (β = -.05, *p* = .303), nor by the interaction between conservatism and task (β = .103, *p* = .101). Because perceived risk is a function of conservatism (although only for highly consequential tasks), these results suggest that conservatism predicts trust, and that this effect is explained by the intermediary effects of conservatism on risk (although only for highly consequential tasks). Indeed, the indirect effect of conservatism on trust, mediated by risk, was significant in the car condition (β = .17, 95% CI = .099 to .248), but not in the music condition (β = .03, 95% CI = -.043 to .096). Finally, the index of moderated mediation was significant (β = -.14, 95% CI = -.243 to -.037).

Using fiscal conservatism instead of social conservatism, plus covariates, the results are as follows. Perceived risk was predicted by task, such that it was higher for AI driving cars (M = 63.72) than for AI recommending music (M = 21.06, *t*(503) = 17.28, *p* < .001). Fiscal conservatism also predicted perceived risk (β = .24, *p* < .001). Furthermore, the interaction between task and conservatism was also significant (β = .23, *p* = .003). Conservatism predicted perceived risk of AI driving a car, such that higher conservatism was associated with higher perceived risk (β = .23, *p* < .001), but conservatism had no effect on the perceived risk of AI recommending music (β = .00, *p* = .992).

We then focus on trust in AI, the dependent variable. Trust was predicted by perceived risk (β = -.63, *p* < .001), but not by task (β = 1.21, *p* = .789), conservatism (β = -.06, *p* = .165), nor by the interaction between conservatism and task (β = .105, *p* = .103). The indirect effect of conservatism on trust, mediated by risk, was significant in the car condition (β = .15, 95% CI = .066 to .236), but not in the music condition (β = .00, 95% CI = -.077 to .069). Finally, the index of moderated mediation was significant (β = -.15, 95% CI = -.271 to -.046).

Using general conservatism instead of social conservatism, plus covariates, the results are as follows. Perceived risk was predicted by task, such that it was higher for AI driving cars (M = 63.72) than for AI recommending music (M = 21.06, *t*(503) = 17.28, *p* < .001). General conservatism also predicted perceived risk (β = .25, *p* < .001). Furthermore, the interaction between task and conservatism was also significant (β = .22, *p* = .010). Conservatism predicted perceived risk of AI driving a car, such that higher conservatism was associated with higher perceived risk (β = .24, *p* < .001), but conservatism had no effect on the perceived risk of AI recommending music (β = .03, *p* = .599).

We then focus on trust in AI, the dependent variable. Trust was predicted by perceived risk (β = -.63, *p* < .001), but not by task (β = 3.00, *p* = .530) or conservatism (β = -.06, *p* = .195). The interaction between conservatism and task was significant (β = -.13, *p* = .049). The indirect effect of conservatism on trust, mediated by risk, was significant in the car condition (β = .16, 95% CI = .070 to .239), but not in the music condition (β = .02, 95% CI = -.057 to .099). Finally, the index of moderated mediation was significant (β = -.14, 95% CI = -.258 to -.028).

**STUDY 4**

In the main text, we report the effects of social conservatism, condition, and their interaction on perceived risk and trust in AI, with age, gender, income, and education as covariates. Here, we report the same analyses without covariates, and similar analyses with covariates but with fiscal and general conservatism instead of social conservatism. First, the same analysis without covariates was as follows.

*Risk.* We observed a significant effect of conservatism, β = 0.21, *p* < .001, a marginal effect of the emotion anthropomorphism condition relative to the control condition, β = -10.88, *p* = .084, and an interaction between conservatism and the emotion anthropomorphism condition, β = .18, *p* = .042. The interaction demonstrates that conservatism had a significant effect on perceived riskiness in the control condition (β = .21, *p* < .001), but no effect in the emotion condition (β = .03, *p* = .707). No other condition interacted with conservatism (risk x conservatism interaction β = .11, *p* = .197; analogy x conservatism interaction β = .08, *p* = .362).

*Trust.* We also observed the same pattern for the trust in AI. We observed a significant effect of conservatism, β = -.27, *p* < .001, a marginal effect of the emotion anthropomorphism condition relative to the control condition, β = 12.77, *p* = .063, and a significant interaction between conservatism and the emotion anthropomorphism condition, β = .19, *p* = .044. Conservatism again had an effect on trust in AI in the control condition (β = -.28, *p* < .001), but not in the emotion condition (β = -.09, *p* = .238). Again, no other condition had a main effect on trust (risk condition β = 9.89, *p* = .137; analogy condition β = -6.79, *p* = .329), nor did they interact with conservatism (risk x conservatism interaction β = .04, *p* = .679; analogy x conservatism interaction β = .11, *p* = .279).

For the same analysis with fiscal instead of social conservatism, the results were as follows.

*Risk.* We observed a significant effect of fiscal conservatism, β = 0.14, *p* = .011, no main effects of any condition, and no interactions between fiscal conservatism and any condition.

*Trust.* We observed a significant effect of conservatism, β = -.18, *p* = .004, a marginal *negative* effect of the analogy condition relative to the control condition, β = -10.18, *p* = .063, and a marginal interaction between conservatism and the analogy condition, β = -.18, *p* = .058. Conservatism had an effect on trust in AI in the control condition (β = -.18, *p* = .006), and a stronger effect in the analogy condition (β = -.35, *p* < .001). No other condition had a main effect on trust (risk condition β = 8.92, *p* = .111; emotion condition β = 2.77, *p* = .638), nor did they interact with conservatism (risk x conservatism interaction β = .03, *p* = .694; analogy x conservatism interaction β = .04, *p* = .633).

Finally, for the same analysis with general conservatism instead of social conservatism, the results were as follows.

*Risk.* We observed a significant effect of fiscal conservatism, β = 0.17, *p* = .004, no main effects of any condition, and no interactions between fiscal conservatism and any condition.

*Trust.* We observed a significant effect of conservatism, β = -.25, *p* < .001, no main effects of any condition, and no interactions between fiscal conservatism and any condition.
